# From baconian to popperian neuroscience

**DOI:** 10.1186/2042-1001-2-2

**Published:** 2012-01-30

**Authors:** David Gamez

## Abstract

The development of neuroscience over the past 50 years has some similarities with the development of physics in the 17th century. Towards the beginning of that century, Bacon promoted the systematic gathering of experimental data and the induction of scientific truth; towards the end, Newton expressed his principles of gravitation and motion in a concise set of mathematical equations that made precise falsifiable predictions. This paper expresses the opinion that as neuroscience comes of age, it needs to move away from amassing large quantities of data about the brain, and adopt a popperian model in which theories are developed that can make strong falsifiable predictions and guide future experimental work.

## Introduction

It is possible to interpret the ways of science more prosaically. One might say that progress can '...come about in only two ways: by gathering new perceptual experiences, and by better organizing those which are available already', but this description of scientific progress, although not actually wrong, seems to miss the point. It is too reminiscent of Bacon's induction: too suggestive of his industrious gathering of the 'countless grapes, ripe and in season', from which he expects the wine of science to flow: of his myth of a scientific method that starts from observation and experiment and then proceeds to theories... The advance of science is not due to the fact that more and more perceptual experiences accumulate in the course of time... Bold ideas, unjustified anticipations, and speculative thought, are our only means for interpreting nature: our only organon, our only instrument, for grasping her. And we must hazard them to win our prize. Those among us who are unwilling to expose their ideas to the hazard of refutation do not take part in the scientific game.

Popper [1], pp. 279-80

Bacon's *Novum Organum *[2] set out a bold agenda for science that started with the systematic gathering of tables of experimental data. He proposed that an inductive method could be applied to the gathered facts to produce more abstract generalizations, and in this way the edifice of scientific knowledge could be built up. Although accurate data are essential to any scientific enterprise, I argue in this opinion piece that the gathering of facts about the brain needs to be complemented by a greater focus on falsifiable theories, which can be tested by experiments and set the agenda for further research. Neuroscience needs to become more popperian if it is to become more scientific.

Developments in neuroscience have led to an incredible expansion of our knowledge about the brain, giving us a broad understanding of the functional specialization of brain areas, a good idea about macro and micro connection patterns, and detailed information about the structure and function of individual neurons. Although this knowledge is essential for neuroscientific progress, there is a tendency for it to be viewed as an end in itself, rather than a prelude to scientific work based on mathematical theories that make falsifiable predictions about the brain.

One problem with an excessive focus on knowledge-gathering is that facts about experimental measurements are often confused with explanations. But the 'lighting up' of the 'language faculty' in a functional magnetic resonance imaging (fMRI) scan does not *explain *how the brain produces language; it just tells us that this part of the brain is more linked (on average) to language production than other parts, which might also be essential. Correlations between brain activity and brain functions need to be explained by a scientific theory.

Some people seem to think that base facts can be transmuted into scientific gold by developing models and matching them to brain measurements. Such a procedure can be a useful starting point for the development of scientific theories, but a model that matches a dataset for a finite period of time is no more of an explanation than the original dataset. There are a potentially infinite number of models that can fit any particular set of data at a given level of approximation (pick your favorite machine-learning algorithm), and so the fact that a model simply matches data is not a useful piece of scientific knowledge. A match between model and data might also be thought to be a sign that the model is describing what the brain is *actually *doing (when it is generating the data), but according to Putman [3], it is senseless to claim that the brain is implementing any particular model or function, because an open physical system can be interpreted as implementing an infinite number of functions. Models that merely *match *datasets are of no use to science; they must be *tested *by making large numbers of falsifiable predictions.

Many people believe that the brain can be understood by developing simulations based on very detailed multi-compartment models [4], point neurons [5], neuronal groups [6] or oscillators [7]. The logical extension of this type of work would be to scan a brain into a computer at high resolution, and connect a simulation based on this data to the original body - potentially producing a complete working simulation of the brain. Although simulation of the brain is a valuable approach that can make limited predictions about its response to perturbation, it is not obvious that a detailed copy of the brain (if it could be done) would give us much idea about how it *works*. It is as if physicists investigating planetary motion were to go into an empty region of space and construct a test solar system out of large quantities of matter. These planetary engineers might eventually get a solar system working, but they would not get any closer to the generalization provided by Newton's equations.

Finally, a neuroscience that limits itself to measurements of the brain cannot be completed because there is a potentially infinite amount of experimental knowledge of this kind. The brain has innumerable facts at different levels, many of which are only starting to be considered by neuroscience, for example, electromagnetic waves [8] or glia activity [9]. We could go on gathering facts forever without ever understanding how the brain works.

Thus far, the negative critique. I will now highlight some areas where the baconian to popperian transition is starting to occur, and brain theories capable of falsifiable prediction are beginning to emerge.

### Global theories of brain function

A number of people have developed explanatory theories of the brain based on simple universal principles. A good example of this approach is Friston [10], who uses the principle of free-energy minimization to explain many aspects of the brain's structure and function, and suggests that this can unify different perspectives on how the brain works. Other examples of global brain theories are neural Darwinism [11] and the bayesian brain hypothesis [12].

This type of theory offers a high-level explanation of the brain that captures many aspects of its operation, and does a neat job of abstracting away from the messiness of neural measurements and circuits. However, this approach has a tendency to focus on explanations of structures and functions that we are already aware of in the brain (for example, Fletcher and Frith's study on schizophrenia [13]), with few attempts to generate predictions that could be tested by new experiments. It is also an open question about how far some of these global theories can be pushed without taking evolutionary hacks and the brain's hard-wired structure into account, and it is not known whether global theories based on relatively simple principles will be capable of making detailed predictions about representational and conscious states, without being complemented by some of the work described in the next two sections.

### Representation

The brain's encoding of information has been the subject of extensive empirical investigation. Information-holding or representational states are typically identified by exposing the brain to a set of stimuli and identifying internal states that co-vary with the presence of the stimuli. For example, Hubel and Wiesel [14] identified neurons in the cat visual cortex, whose firing changed when the animal was exposed to a bar of light moving in a particular direction, and electrode implantation work in humans has shown that neurons can encode information about individual people [15]. There has also been a substantial amount of related work on 'brain reading', which uses statistical correlations between properties of the stimulus and fMRI data to make predictions about different types of mental content [16].

**Figure 1:**
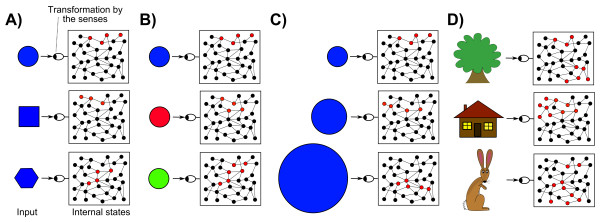
**Standard technique for identifying representational mental states**. **(A) **system is exposed to different shapes and its response measured; **(B, C) **the circle response is characterized more precisely by exposing the system to circles with different colors and sizes; **(D) **eventually it might be possible to identify the system's responses to more complex stimuli.

The main limitation of this method for identifying representations is that there are an extremely large number of properties of a given stimulus to which the system could be responding, which reach unmanageable proportions as the complexity of the system increases. For example, if a system produces a response to a blue circle, then this could be representing the colour of the circle, the size of the circle, the time at which the circle appears and so on, and a laborious and possibly infinite series of tests have to be performed to precisely identify the representational content (Figure 1). Further problems lie in the fact that the brain's learning makes each person's representations different, and the whole process has to be repeated for each new brain and for each brain architecture.

A better approach would be to move away from measuring the parts of the brain that respond to different types of information, and instead develop a theoretical understanding of the transformation process that occurs when data enter the senses, and the further transformations that take place within the brain. For example, instead of measuring the response of neurons in the visual cortex to bars of light, we could use knowledge about the anatomy of the retina to develop systematic accounts of representation that explain how information moves from the high-dimensional space of the world into the second high-dimensional space of spikes in the optic nerves, leading to mathematical or information-theoretic accounts that can be used to identify representational states immediately downstream of the senses. This would avoid the combinatorial problems associated with probing a system for representations, and enable us to make predictions about the representational contents of the brain by reconstructing its environment from knowledge about the active representational states and the senses, possibly using three-dimensional graphics to visualize the representational contents. Some of the more promising work in this area is already moving from correlation measurements to a modeling approach capable of predictions [17].

### Consciousness

What's the matter with consciousness, then, and how should we proceed? Early on, I came to the conclusion that a genuine understanding of consciousness is possible only if empirical studies are complemented by a theoretical analysis. Indeed, neurobiological facts constitute both challenging paradoxes and precious clues to the enigma of consciousness. This state of affairs is not unlike the one faced by biologists when, knowing a great deal about similarities and differences between species, fossil remains, and breeding practices, they still lacked a theory of how evolution might occur. What was needed, then as now, were not just more facts, but a theoretical framework that could make sense of them.

Tononi [18], p. 217

Research on the neural correlates of consciousness attempts to identify the minimal neuronal mechanisms that are jointly sufficient for any one specific conscious percept [19]. To date, this type of work has identified parts of the brain and dynamic aspects of neural activity that seem to be linked to conscious information-processing. These data about the neural correlates of consciousness are important, but data-gathering cannot continue *indefinitely*, nor can it continue *blindly*. We need to develop mathematical and algorithmic theories of consciousness that can make falsifiable predictions about phenomenal states and set the agenda for future research.

This type of work has already started, and a number of mathematical and algorithmic theories have been developed that could potentially explain the difference between conscious and unconscious information processing; for example, information integration [18], causal density [20] and liveliness [21]. Preliminary experimental work has also been carried out to test the predictions made by these theories [22,23]. However, a great deal of theoretical and experimental work still needs to be performed in this area; some of the current approaches have severe performance limitations, others require further refinement, and many theories of consciousness lack formal definitions and have never been tested. Eventually a theory-driven mathematical approach might be able to move beyond facts about correlations, and generate detailed predictions about the brain's phenomenology that can be compared with first-person reports.

### Some possible objections

#### Measurement limitations make it impossible to test theories

Perhaps our predictions cannot be tested until we can measure all of the neurons' states in real time? One problem with this objection is that it cannot be assumed that neuron firing is the correct level of abstraction; spikes are just one set of brain measurements that we can make. Ion channels or local field potentials might turn out to be the best starting point for explanatory theories. A second problem with this objection is that it is possible (perhaps probable) that a higher level of abstraction, such as oscillators modeling neuron groups [7], will be the most productive level at which an understanding of the brain can be reached. Newton did not have access to the state of every molecule in every planet, and yet his equations could predict the planetary bodies' future movements with a high degree of accuracy. Finally, although the available data constrain our ability to test theories, a good theory should be capable of making predictions that can be tested with our current technology.

#### Newton was wrong

Newton's laws had major flaws, failed to account for the precession of the perihelion of Mercury, and should not be held up as a paradigmatic example of scientific truth. While Newton's equations failed to be the final answer, they are a beautiful example of a theory that makes strong falsifiable predictions which can be experimentally tested.

#### The dynamic complexity of the brain makes accurate prediction impossible

The brain *is *a complex dynamical system, but so are the planets: both can be highly stable or highly sensitive to their initial conditions. We have mathematical techniques for analyzing and describing dynamic systems, and so it might be possible to explain how the complex behaviour of the brain arises from a simple set of interacting principles. The extent to which the brain can be mathematically described is an empirical question.

#### This work is already being carried out

This article has highlighted some of the theories about the brain that are capable of making falsifiable predictions, and large numbers of mathematical models of different aspects of neural circuits have been developed. I welcome the valuable work that is being done on the development of strong falsifiable theories, and encourage more researchers to take this approach and test their theories in the laboratory.

#### Bacon was great

Yes, Bacon was great: many aspects of his method are true and useful, and he cleared out a lot of Aristotelian rubbish. However, in my opinion, Popper provides a much more accurate description of the ideal scientific method.

#### Popper was wrong

Some would argue that Popper presents an outmoded account of the philosophy of science, which should be replaced by Kuhn [24] at least, or perhaps Feyerabend [25] or Latour [26]. Some of these later 'relativist', 'constructivist', 'post-modern' accounts reject the possibility of scientific progress altogether. Insofar as neuroscience understands itself as engaged in an enterprise to scientifically understand the brain, it needs a model of what science is, and I would argue that Popper provides a carefully thought out and convincing account of what good scientific practice should be. Other philosophies of science can be used to interpret neuroscience, but many of them are considerably less useful as guiding principles than Popper: how (or why) would one actively pursue a neuroscience based on Feyerabend or Latour?

### The way ahead

We are far too closely bound to the language of measurement (spikes, local field potentials, haemodynamic responses, and so on). New ways of describing brain activity are required that are more easily expressed in a mathematical form; we need something along the lines of Newton's mass (a more abstract way of understanding the measured weight of a body). Much promising work has been carried out in computational neuroscience [27,28] that could be taken further, and greater use could be made of category theory, which has already been used to describe biological systems and the brain at different levels of abstraction [29,30]. Information theory has been applied to the science of consciousness, and a number of mathematical methods can be used to quantify functional and effective connectivity, such as mutual information, Granger causality, and transfer entropy [31]. These more abstract descriptions of the brain can be used to develop mathematical and algorithmic theories that can predict the brain's behaviour and its representational and conscious states. These predictions can be compared with experimental measurements and behavioral reports; bad theories can be discarded, and good theories retained (more detailed suggestions about the way ahead can be found in my previous work [32]).

## Conclusions

This opinion piece has not in any way intended to diminish the large amount of extremely useful work that is being carried out in neuroscience. However, I have tried to highlight the fact that a significant proportion of the science in 'neuroscience' has a more baconian than popperian character, with brain measurement being seen almost as an end in itself, rather than as a starting point for the development of falsifiable theories. We need to change emphasis and priorities - move beyond measurements of the brain to mathematical models that make many strong predictions which can be experimentally tested. These models should not be fitted to a particular dataset, but based on general laws that could in principle be applied to intelligent creatures with different neuroanatomy, such as birds or octopi, and possibly to artificial systems as well. Although this presents considerable challenges, I have touched on some promising work that is already moving in this direction.

## Competing interests

The author declares that he has no competing interests.

## Authors' contributions

This article was written by (and is the opinion of) DG.

## Author's Information

DG holds PhDs in both philosophy and computer science. One of his main areas of research is how a science of human and machine consciousness can be developed based on mathematical and algorithmic theories. He is currently working as a research associate/postdoctoral researcher at the Department of Computing, Imperial College London, where he is carrying out practical and theoretical work on the control of robots using biologically inspired spiking neural networks.
